# Necroptosis–senescence crosstalk in tubulointerstitial fibrosis: the mtDNA–cGAS–STING–PFKFB3 axis as a metabolic bridge

**DOI:** 10.3389/fmed.2026.1882589

**Published:** 2026-09-03

**Authors:** Donghui Wang, Hongxiang Zheng, Xinyue Zhang, Chenghua Zhang, Dandan Zhao, Shuqi Min, Shenju Wang

**Affiliations:** 1 Graduate School, Nanjing University of Chinese Medicine, Nanjing, China; 2 Department of Nephrology, Changzhou Affiliated Hospital of Nanjing University of Chinese Medicine, Changzhou, China

**Keywords:** cellular senescence, cGAS-STING, chronic kidney disease, glycolysis, mitochondrial DNA, necroptosis, PFKFB3, tubulointerstitial fibrosis

## Abstract

**Background:**

Chronic kidney disease progresses irreversibly toward tubulointerstitial fibrosis. Although renin-angiotensin system inhibitors and sodium-glucose cotransporter 2 inhibitors slow progression, they cannot block or reverse fibrosis. Recent work shows that necroptosis and cellular senescence in tubular epithelial cells are not independent events; they form a dynamically evolving continuum through the mitochondrial DNA-cyclic GMP-AMP synthase-stimulator of interferon genes-interferon regulatory factor 3-6-phosphofructo-2-kinase/fructose-2,6-bisphosphatase 3-glycolysis axis.

**Methods:**

We conducted a narrative review of peer-reviewed literature at the intersection of necroptosis, cellular senescence, and renal tubulointerstitial fibrosis. Systematic searches of PubMed/MEDLINE, Web of Science, and Embase were performed on 15 July 2026 using Medical Subject Headings terms and free-text keywords; evidence was classified using a pragmatic taxonomy (*in vitro*, *in vivo*, phase I/II, and *post hoc*/subgroup analyses).

**Results:**

Necroptotic tubular epithelial cell death releases mitochondrial DNA that activates cyclic GMP-AMP synthase-stimulator of interferon genes-interferon regulatory factor 3-dependent 6-phosphofructo-2-kinase/fructose-2,6-bisphosphatase 3 transcription, driving glycolytic reprogramming that sustains and amplifies the senescence-associated secretory phenotype through histone H4 lysine 12 lactylation. Senescence-associated secretory phenotype factors activate interstitial fibroblasts and reciprocally prime adjacent tubular epithelial cells for necroptosis, creating a self-amplifying loop. Existing targeted strategies—inhibitors of receptor-interacting serine/threonine-protein kinase 1, senolytics, 6-phosphofructo-2-kinase/fructose-2,6-bisphosphatase 3 blockers, and multi-target traditional Chinese medicine—show preclinical efficacy but face translation barriers arising from target selectivity, biomarker absence, and ill-defined therapeutic windows.

**Conclusion:**

Future antifibrotic strategies should shift from single-molecule inhibition to network remodeling, using chronology-based combination therapy tailored to disease stage and guided by dynamic biomarkers.

## Graphical abstract

**Figure:**
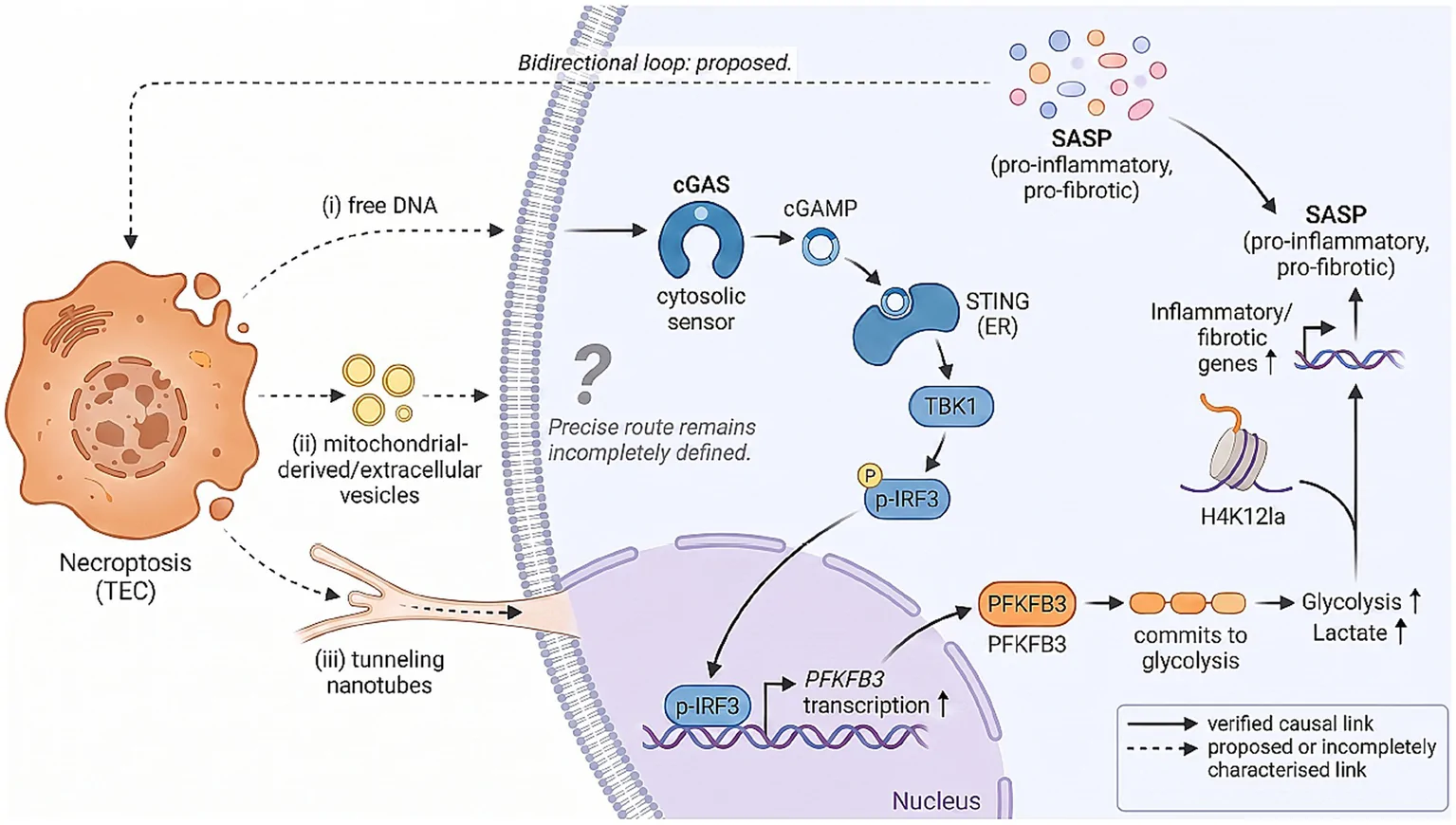
The central hypothesis: mtDNA–cGAS–STING–PFKFB3–glycolysis axis. Necroptotic death of tubular epithelial cells releases mitochondrial DNA (mtDNA) into the extracellular space. The precise route by which extracellular mtDNA crosses the plasma membrane of recipient tubular epithelial cells remains incompletely defined; plausible mechanisms include uptake via extracellular vesicles, phagocytosis or macropinocytosis of naked DNA, and cGAMP-mediated bystander propagation, although direct renal evidence is still limited. Once in the cytosol, mtDNA activates the cytosolic DNA sensor cGAS. cGAS synthesizes cGAMP, which activates STING on the endoplasmic reticulum. Activated STING recruits TBK1 to phosphorylate IRF3, which translocates to the nucleus and drives transcription of PFKFB3. PFKFB3 catalyzes the synthesis of Fru-2,6-BP, the most potent allosteric activator of phosphofructokinase-1 (PFK1), thereby committing the cell to glycolysis even under aerobic conditions (Warburg-like metabolism). Glycolytic flux generates lactate, which drives histone H4K12 lactylation (H4K12la) at NF-κB target gene promoters, locking in a pro-inflammatory and pro-fibrotic transcriptional program. The resulting SASP feeds back to promote further necroptosis in adjacent cells, establishing a self-sustaining loop. Evidence status: While the release of mtDNA during TEC necroptosis (step 1) and its downstream activation of cGAS–STING–PFKFB3 (steps 3–5) are supported by direct renal experimental evidence, the intercellular transit of mtDNA from necroptotic TECs to neighboring TECs (step 2) remains a working hypothesis extrapolated from non-renal systems and should not be interpreted as a proven causal step (extracellular vesicles, phagocytosis, and tunneling nanotubes).

## 1 Introduction: from programmed necrosis to the senescence microenvironment

Chronic kidney disease affects roughly 8–10% of adults worldwide, and tubulointerstitial fibrosis—not glomerular disease—is the common final pathway driving progressive renal function loss. Regardless of whether the initial insult stems from diabetes, hypertension, autoimmune disease, or ischaemia–reperfusion injury, abnormal extracellular matrix deposition in the renal interstitium leads to irreversible nephron loss. Existing standard therapies slow the disease but do not stop or reverse fibrosis. Novel strategies that target fibrotic mechanisms directly remain an unmet need (1, 2).

The cellular landscape of renal fibrosis has been revolutionized by single-cell and spatial multi-omics. “Maladaptive tubules” were identified by Abedini et al., which are epithelial cells that acquire active secretory functions and spatially overlap with sites of fibroblast activation (3). Recent reviews have summarized the broader mechanistic landscape from inflammation to therapeutics (2). Hinze et al. found that injured tubular epithelial cells have a repertoire of potential fates: adaptive repair, maladaptive repair, senescence, and death (4). These cells are not passive victims but rather act as signal hubs, converting acute injury into chronic fibrotic programs through crosstalk with macrophages, fibroblasts, and endothelial cells.

At the center of this communication network lies the convergence of necroptosis and cellular senescence. Necroptosis, driven by the receptor-interacting serine/threonine-protein kinase 1 (RIPK1)–RIPK3–mixed lineage kinase domain-like pseudokinase (MLKL) axis, has been viewed as acute programmed cell death characterized by membrane rupture and DAMP release. Senescence, marked by p16/p21 and senescence-associated secretory phenotype (SASP), has been seen as a chronic cell-cycle arrest. Evidence now shows these fates are not sharply separated. During necroptosis, released damage-associated molecular patterns (DAMPs)—especially mtDNA—activate cGAS–STING signaling, while PFKFB3 upregulation stabilizes the senescent phenotype and amplifies SASP (5, 6). SASP factors can reciprocally activate necroptotic signals, forming a self-sustaining loop.

**Figure 1:**
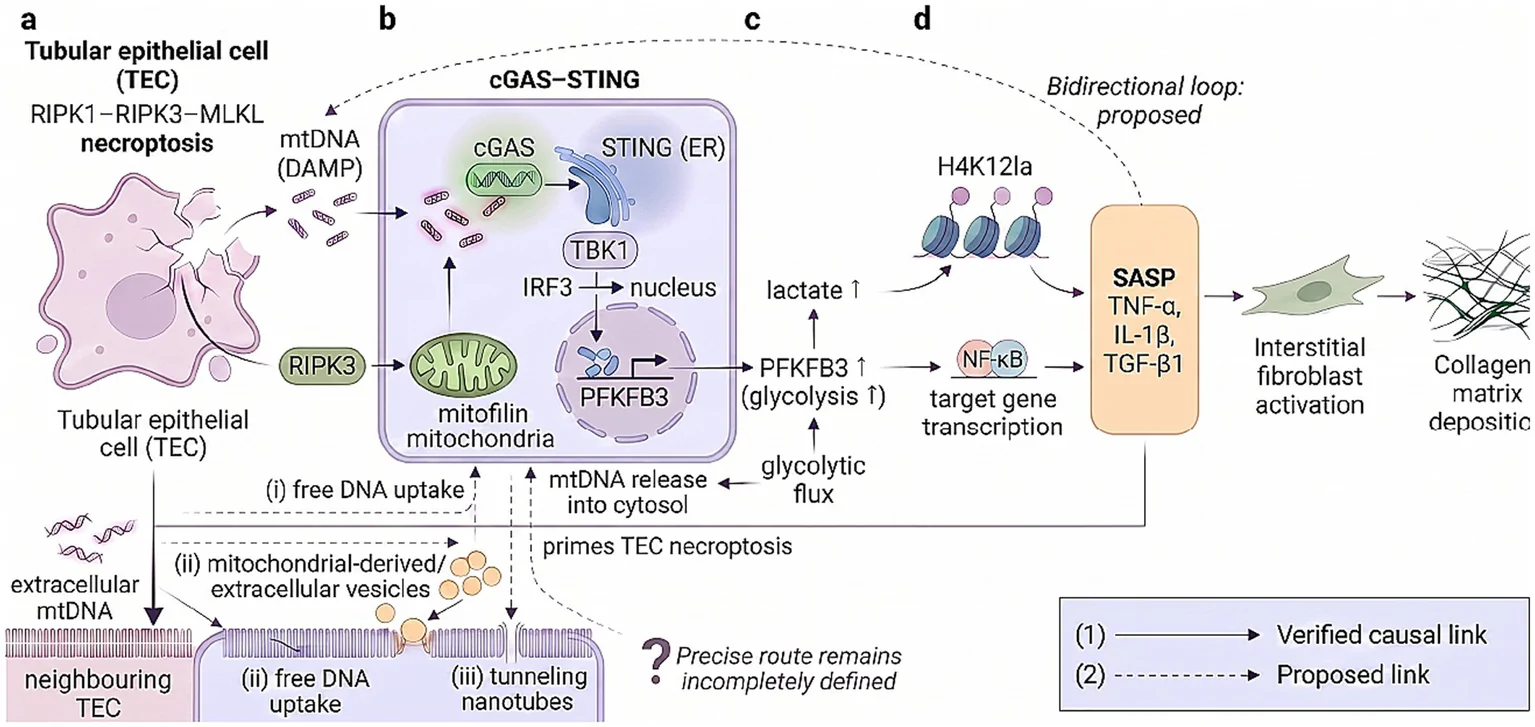
The necroptosis–senescence–fibrosis axis in chronic kidney disease. Solid arrows indicate experimentally supported causal relationships; dashed arrows indicate proposed or incompletely characterized links. **(a)** Acute injury triggers RIPK1–RIPK3–MLKL-dependent necroptosis in tubular epithelial cells (TECs), culminating in plasma membrane rupture and release of damage-associated molecular patterns (DAMPs), including mitochondrial DNA (mtDNA). **(b)** Extracellular mtDNA is proposed to be internalized by neighboring TECs through mechanisms that remain incompletely defined (working hypothesis extrapolated from non-renal systems) (free DNA, mitochondrial-derived vesicles, or extracellular vesicles); Once in the cytosol, mtDNA activates the cGAS–STING pathway. STING-dependent IRF3 nuclear translocation drives PFKFB3 transcription, inducing glycolytic reprogramming. **(c)** Glycolytic lactate promotes histone lactylation (H4K12la) and sustains NF-κB-dependent SASP expression. **(d)** SASP factors (TNF-*α*, IL-1β, and TGF-β1) activate interstitial fibroblasts and may reciprocally prime adjacent TECs for necroptosis, constituting a bidirectional positive feedback loop that remains a working hypothesis. The progressive expansion of the fibrotic niche is driven by verified paracrine activation and proposed reciprocal reinforcement.

This narrative review proposes that mtDNA release from TEC necroptosis activates interferon regulatory factor 3 (IRF3)-dependent PFKFB3 transcription via cGAS–STING, driving glycolytic reprogramming that in turn drives TEC senescence and SASP-dependent interstitial fibrosis. Compared with existing reviews, we treat metabolic reprogramming—not pure signal transduction—as the bridge between acute death and chronic fibrosis, and we examine the structural roots of translational failure from the perspectives of target selectivity, biomarker absence, and temporal dynamics. The central hypothesis is illustrated in Figure 1.

## 2 Methods

Search strategy and selection criteria. We conducted a narrative review of peer-reviewed literature at the intersection of necroptosis, cellular senescence, and renal tubulointerstitial fibrosis. Systematic searches of PubMed/MEDLINE, Web of Science, and Embase were performed on 15 July 2026. We combined Medical Subject Headings (MeSH) terms and free-text keywords related to necroptosis (e.g., “necroptosis,” “RIPK1,” “RIPK3,” “MLKL”), cellular senescence (e.g., “cellular senescence,” “senescence-associated secretory phenotype,” “SASP”), kidney fibrosis (e.g., “kidney fibrosis,” “tubulointerstitial fibrosis,” “chronic kidney disease”), and metabolic signaling (e.g., “PFKFB3,” “cGAS–STING,” “mitochondrial DNA”). Equivalent strategies were adapted for Web of Science (topic-field searches) and Embase (Emtree terms). No language restriction was applied; English-language articles were prioritized. Bibliographies of included reviews and meta-analyses were manually screened for additional primary studies.

Study selection and data extraction. Two authors (D.W. and H.Z.) independently screened titles and abstracts, with disagreements resolved by consensus or arbitration by a third author (S.W.). Inclusion criteria: (i) original articles, reviews, or mechanistic studies investigating necroptosis and/or cellular senescence in renal tubular epithelial cells, interstitial fibroblasts, or myeloid cells in the context of chronic kidney disease (CKD), acute kidney injury (AKI)-to-CKD transition, diabetic kidney disease, hypertensive nephropathy, obstructive uropathy, or ischaemia–reperfusion injury; (ii) studies reporting molecular mechanisms, genetic or pharmacological perturbations, single-cell/spatial transcriptomics, or preclinical therapeutic interventions. Exclusion criteria: case reports, conference abstracts, non-peer-reviewed preprints, and studies in which kidney fibrosis was not a primary outcome or in which necroptosis and senescence were studied exclusively in non-renal cell types. Data were extracted into a standardized form (first author, year, model, intervention, target, main findings, and limitations) and synthesized narratively; no formal quality scoring or statistical meta-analysis was performed. For animal studies, we recorded randomization, blinding, and sample-size reporting according to the ARRIVE guidelines 2.0. A standardized data-extraction form is available from the corresponding author upon request.

Evidence assessment. Because the included literature spans mechanistic studies, preclinical efficacy experiments, and early-phase clinical trials, we did not apply a single uniform quality-appraisal tool. Instead, evidence was classified using a pragmatic taxonomy: *in vitro* observations; *in vivo* genetic or pharmacological studies in animal models; Phase I/II clinical trials; and post-hoc or subgroup analyses of larger trials. This grading is reported in the “Level of evidence” columns of Tables 1–3. Within each category, we considered genetic rescue, pharmacological inhibition, and human tissue correlation as stronger support than observation alone.

**Table 1:** Key preclinical evidence for necroptosis in kidney disease.

Model	Intervention	Key finding	Ref	Level of evidence
Unilateral IRI (mouse)	Ripk3^−/−^ or Mlkl^−/−^	Reduced tubular necrosis, macrophage infiltration, NLRP3 activation, and long-term interstitial fibrosis	(10)	Preclinical (*in vivo*)
5/6 subtotal nephrectomy (rat)	Necrostatin-1	Decreased BUN, serum creatinine, and tubular injury score; necroptosis exceeded apoptosis as the dominant death mode	(11)	Preclinical (in vivo)
Diabetic kidney disease (mouse/cell)	RIPK1 inhibitor	Attenuated tubular necroptosis and inflammation; RIPK3–PGAM5–Drp1 axis drove mitochondrial dysfunction in podocytes	(12, 14)	Preclinical (in vivo)
Unilateral IRI (mouse)	Nec-1s or GSK’872 (days 3–9)	Reduced chronic kidney injury and fibrosis at day 28, but effect was partial/delayed rather than fully preventive	(13)	Preclinical (in vivo)
Renal IRI (mouse)	Genetic/pharmacological RIPK3 inhibition	RIPK3 translocated to mitochondria, promoted mtDNA release via mitofilin, and activated cGAS–STING	(16)	Preclinical (in vivo)

**Table 2:** Comparison of targeted therapeutic strategies in renal fibrosis.

Target	Representative agent	Mechanism	Major limitation	Level of evidence
RIPK1	Necrostatin-1s, GSK2982772	Blocks necrosome assembly and MLKL activation	Narrow therapeutic window; potential immunosuppression	Phase I/II
RIPK3	GSK’872	Inhibits MLKL phosphorylation	Apoptotic off-target effects at high dose	Preclinical
MLKL	None available	Prevents membrane rupture and DAMP release	Lack of druggable pocket; no clinical candidate	Preclinical
Senescent TEC	ABT-263 (navitoclax), D + Q	Senolytic clearance of p16^+^/p21^+^ cells	Risk of removing transiently beneficial repair-phase senescent cells	Phase I/II
SASP suppression	Metformin, rapamycin	Senomorphic dampening of NF-κB/mTORC1-dependent secretion	Incomplete suppression; requires chronic dosing	Preclinical
PFKFB3	3PO, PFK158	Blocks glycolytic flux and histone lactylation	Systemic metabolic toxicity; need for kidney-specific delivery	Preclinical
HIF-1α	FG-4592 (stabilizer)	Modulates hypoxic adaptation	Narrow temporal window; chronic activation worsens fibrosis	Preclinical
Mitophagy	Urolithin A	Promotes PINK1/Parkin-dependent clearance	Limited human PK data; variable natural product content	Preclinical

**Table 3:** Structural lessons from failed clinical trials in renal fibrosis.

Agent	Target	Indication	Phase	Outcome	Key lesson	Level of evidence
Lademirsen	miR-21	Alport syndrome	II	Terminated early for futility	Single-target disruption is insufficient in a compensatorily redundant fibrotic network	Phase II
Fresolimumab	TGF-β	FSGS, DKD	II	No eGFR benefit vs. placebo	Blocking one master cytokine triggers pathway bypass; trial design lacked molecular stratification	Phase II
Bardoxolone methyl	Nrf2	DKD (BEACON)	III	Stopped for cardiovascular events and mortality	Broad pleiotropic activation disrupts vascular homeostasis; eGFR surrogate masked true risk	Phase III

## 3 Molecular mechanisms of tubular necroptosis and renal pathology

Necroptosis depends on the RIPK1–RIPK3–MLKL signaling cascade. TNF-*α* binding to TNFR1 normally activates NF-κB pro-survival pathways; when caspase-8 is blocked, RIPK1 and RIPK3 assemble the “necrosome” through their RHIM domains. Activated RIPK3 phosphorylates MLKL at specific residues—Ser345 in mice and Thr357 and Ser358 in humans—triggering a marked conformational change (7). Meng et al. used cryo-EM and X-ray crystallography to show that human RIPK3 maintains MLKL in a folded inactive conformation through a unique molecular clamp; only when RIPK3 itself is activated does this clamp release, allowing MLKL to initiate oligomerization (8). The liberated MLKL exposes its N-terminal four-helix bundle, which inserts into lipid bilayers and disrupts membrane integrity.

Samson et al. tracked MLKL translocation by live-cell imaging and found that MLKL oligomers first form in the perinuclear region, then migrate toward the plasma membrane via microtubule-dependent transport; accumulation must exceed a critical threshold to trigger rupture (9). This threshold mechanism means necroptosis is quantitatively regulated by MLKL expression, phosphorylation efficiency, and membrane trafficking rate. In renal ischaemia–reperfusion models, the key activation window lies between 3 and 12 h after reperfusion.

### 3.1 Pathological evidence for necroptosis in kidney disease

Pathological evidence spans acute and chronic models. In ischemia–reperfusion injury, Ripk3 or Mlkl knockout reduces tubular necrosis, macrophage infiltration, and NOD-like receptor family pyrin domain-containing 3 (NLRP3) inflammasome activation, attenuating long-term fibrosis (10). In the 5/6 subtotal nephrectomy model, necroptotic death exceeds apoptosis, and Necrostatin-1 ameliorates tubular injury scores (11). In diabetic kidney disease, high glucose and free fatty acids synergistically induce RIPK3 and MLKL phosphorylation in tubular epithelial cells (12). Pefanis et al. found that RIPK1 or RIPK3 inhibition protects acutely and subacutely, but the effect on chronic fibrosis is more “delayed” than “prevented”—suggesting necroptosis is the trigger, not the sustainer, of fibrosis (13). Beyond the tubulointerstitium, RIPK3 drives mitochondrial dysfunction and albuminuria in diabetic podocytopathy through PGAM5-Drp1 signaling (14).

The renoprotective effect of RIPK1 inhibitors supports the causal role of necroptosis. Pefanis et al. evaluated Necrostatin-1s and GSK’872 in unilateral renal IRI: Nec-1s given before ischemia or 15 min after reperfusion reduced acute injury; continuous administration from days 3–9 attenuated fibrosis at day 28. However, the blocking effect on chronic fibrosis was relatively limited compared with the acute effect, implying that once the fibrotic program initiates, simply inhibiting upstream death signals may be insufficient (13).

Beyond MLKL’s role as the terminal necroptosis effector, non-necroptotic functions have been reported in liver and neural models, including vesicle secretion and membrane repair. No direct evidence currently supports such functions in kidney disease; all existing renal studies position MLKL as the executor of necroptosis. Whether MLKL possesses non-lethal functions in tubular epithelial cells remains theoretical speculation. These context-dependent functions are consistent with the emerging view that MLKL trafficking and accumulation at the plasma membrane control the kinetics and threshold for necroptosis (9, 15).

### 3.2 DAMP release and necroinflammation

The lytic membrane rupture in necroptosis releases DAMPs, triggering sterile inflammation. Chen et al. demonstrated the activation of the NLRP3 inflammasome in macrophages by supernatant from necroptotic TECs with ASC speck formation and caspase-1-dependent IL-1β release, whose association is abrogated by Ripk3 or Mlkl knockout (10). HMGB1 activates NF-κB through TLR4/RAGE; ATP activates the P2X7 receptor and promotes NLRP3 assembly; mtDNA activates TLR9 and cGAS. Feng et al. discovered that under ischemic stress, RIPK3 translocates to mitochondria and interacts with the inner membrane protein mitofilin, promoting mtDNA release from the matrix into the cytoplasm. The released mtDNA subsequently activates cGAS–STING, driving transcription of type I interferons and pro-inflammatory genes (16). How the released mtDNA reaches the cytosol of neighboring TECs is not established; in non-renal systems, extracellular mtDNA can be internalized via vesicle fusion/endocytosis, scavenger receptor-mediated uptake, or phagocytosis, and cGAMP can propagate the signal independently through gap junctions or anion channels (17–20). This finding directly links the core necroptosis signaling molecule RIPK3 to mtDNA release and provides the upstream trigger for the metabolic reprogramming axis.

These mechanisms constitute a self-amplifying positive-feedback loop: necroptosis releases DAMPs, activating macrophages; activated immune cells release TNF-*α*, IL-1β, and reactive oxygen species (ROS), promoting further necroptosis; new cell death releases more DAMPs, amplifying the response. Chen et al. (10) described this as an “auto-amplification loop” and showed that Ripk3 or Mlkl knockout interrupts it (10). Key preclinical findings are summarized in Table 1.

## 4 Molecular crosstalk between necroptosis and tubular senescence

Necroptosis and senescence were once treated as separate processes. They are not. DAMPs released during necroptosis—especially mtDNA—activate cGAS–STING in neighboring cells, driving metabolic reprogramming and senescence; SASP factors reciprocally amplify necroptosis, forming a bidirectional loop.

### 4.1 From DAMPs to paracrine senescence: the legacy of acute injury

During necroptosis, HMGB1, ATP, mtDNA, and S100 proteins flood the interstitium. HMGB1 is itself an early SASP component, detectable in secretion 24–48 h after radiation-induced senescence, and can induce paracrine senescence in neighboring TECs (21). ATP activates NLRP3 and releases IL-1β, another core SASP factor; its persistent presence further stabilizes the senescent phenotype of neighboring cells. mtDNA is uniquely positioned: it is the only bacterial-origin genomic DNA in the cell, rich in CpG islands, and can be dually recognized by TLR9 and cGAS. Circulating mtDNA levels rise with age and correlate positively with inflammatory cytokines in plasma from elderly individuals (21). It is the shared alarm molecule connecting acute injury with chronic fibrosis. Whether this extracellular mtDNA enters neighboring TECs primarily as free DNA, as vesicle-encapsulated cargo, or via tunneling nanotubes is still unresolved; once inside, endosomal escape appears necessary for cGAS recognition (17, 22, 23). Cellular senescence is also prominent after ischaemia-reperfusion injury, linking acute tubular damage to maladaptive repair and chronic fibrosis (24).

### 4.2 The mtDNA–cGAS–STING axis: a core hub linking necroptosis and senescence

cGAS senses cytosolic DNA and synthesizes cGAMP, which activates STING to recruit TBK1 and IKKε, phosphorylating IRF3 and NF-κB. In senescent cells, cytosolic DNA accumulates from three main sources: mtDNA leakage due to mitochondrial dysfunction, fragmented products after nuclear DNA double-strand breaks, and derepressed endogenous retroelements such as LINE-1. López-Otín et al. noted that transcriptional derepression of LINE-1 is a universal feature of cellular senescence, and its retrotranscription products entering the cytoplasm can directly activate cGAS–STING (21). Zhong et al. showed that declining mitophagy in aged macrophages causes mtDNA leakage and cGAS–STING-driven type I interferon responses (25). Cytoplasmic chromatin fragments released from senescent or dying cells can trigger sterile inflammation independently of infection, bridging nuclear instability to chronic inflammation (26), and persistent cGAS–STING signaling remodels immune system function and reinforces the senescent secretory phenotype during aging (27).

In the kidney, Ha et al. provide the most direct experimental support. Using PTEN-induced putative kinase 1 (PINK1) knockout mice—a model with marked mitochondrial dysfunction due to defective mitophagy—they found that cGAS and STING expression in the kidneys of 24-month-old PINK1−/− mice was significantly higher than in age-matched wild-type mice, and this activation positively correlated with renal interstitial fibrosis, tubular injury scores, and SASP factor expression. *In vitro*, HKC-8 cells treated with PINK1 siRNA and H_2_O_2_ combination showed significant activation of cGAS–STING; the STING inhibitor H-151 was able to reduce these senescence markers (6). As a key regulator of mitophagy, PINK1 deficiency leads to impaired clearance of damaged mitochondria, mtDNA leakage, and cGAS–STING activation—a causal chain validated in kidney aging models.

Importantly, cGAS–STING signaling is not exclusively pathogenic in the kidney. In ischemic preconditioning and certain infection models, STING-dependent type I interferon responses can promote tubular repair and antimicrobial defense. The net effect therefore depends on the intensity and duration of activation: transient, low-level signaling may be adaptive, whereas chronic, high-intensity signaling drives senescence and fibrosis. This duality should inform the design of STING-targeted therapies in CKD.

A 2025 Nature Communications study provided a systemic perspective. Researchers induced persistent, nonmutagenic DNA double-strand breaks in mouse proximal tubular epithelial cells and found that PTEC DNA damage elevated local cGAS and STING and triggered systemic upregulation of SASP factors and senescence markers in liver and visceral adipose tissue. Metabolomic analysis showed these PTECs exhibited mitochondrial dysfunction: basal oxygen consumption and ATP production decreased, fatty acid oxidation was impaired, mtDNA copy number was reduced, and cells were forced toward glycolysis (28). This reveals that PTEC-level mtDNA–cGAS–STING activation can produce whole-body metabolic consequences.

The pathways for mtDNA entry into the cytoplasm differ between acute and chronic contexts. In necroptosis, RIPK3 translocates to mitochondria and interacts with mitofilin, promoting acute, massive mtDNA release accompanied by complete plasma membrane rupture (16). In chronic senescence, mtDNA leakage is more attributable to mitophagy dysfunction or aberrant opening of the mitochondrial permeability transition pore. These two sources do not differ chemically, but their release kinetics differ: necroptosis produces acute, high-intensity release, whereas senescence produces chronic, low-intensity leakage in living cells whose membranes remain intact. Moreover, transit of mtDNA between cells is now viewed as a distinct regulatory node: extracellular mtDNA can be stabilized by vesicles or DNA-binding proteins, and its uptake by recipient cells may occur through endocytosis, phagocytosis, or direct intercellular conduits, with endosomal escape representing a final barrier to cGAS activation (19, 20, 22).

### 4.3 cGAS–STING → IRF3 → PFKFB3 → glycolysis: the molecular chain of metabolic reprogramming

Activation of cGAS–STING is not limited to inflammatory cytokine transcription. Multiple studies have revealed its direct role in metabolic reprogramming, especially glycolytic regulation. Jiang et al. provide key evidence for the kidney. In hypoxia-induced renal fibrosis, they observed significant upregulation of cGAS, STING, and IRF3 in HK-2 cells and IRI mouse kidneys; the cGAS–STING–IRF3 axis directly binds the PFKFB3 promoter, driving transcriptional activation of this key glycolytic rate-limiting enzyme. PFKFB3 catalyzes the conversion of fructose-6-phosphate to fructose-2,6-bisphosphate (Fru-2,6-BP), the most potent allosteric activator of phosphofructokinase-1 (PFK1). The kinase/phosphatase activity ratio of PFKFB3 is approximately 700:1, meaning it almost unidirectionally drives increased glycolytic flux (5). This causal chain was confirmed by siRNA knockdown of cGAS or STING and functional inhibition of IRF3.

PFKFB3 operates across multiple renal cell types. In tubular epithelial cells, Jiang et al. confirmed cGAS–STING directly upregulates PFKFB3 (5). In interstitial fibroblasts, TGF-β1 stimulation induces PFKFB3, and pharmacological inhibition (3PO) or genetic knockdown attenuates αSMA expression and collagen secretion (29). In bone-marrow-derived myeloid cells, conditional Pfkfb3 knockout attenuates renal fibrosis in the UUO model (30).

Wang et al. reported that PFKFB3 was involved in epigenetic regulation. They found that PFKFB3 deficiency significantly alleviated renal interstitial fibrosis in UUO and folic acid nephropathy models in proximal tubular cell-specific Pfkfb3 knockout mice. Mechanistically, PFKFB3-driven glycolytic enhancement produces lactate, which deposits on lysine 12 of histone H4 (H4K12la); enrichment of H4K12la in NF-κB target gene promoter regions promotes transcription of pro-inflammatory and pro-fibrotic genes. PTC-specific Pfkfb3 knockout reduced renal H4K12la levels and decreased NF-κB activity (31). This provides a direct link between metabolic reprogramming and epigenetic regulation in tubular senescence.

Another important regulator of PFKFB3 expression is hypoxia-inducible factor-1α (HIF-1α). Under hypoxic conditions, HIF-1α binds to hypoxia-response elements within the PFKFB3 promoter. In diabetic kidney disease, SAE1-mediated SUMOylation of HIF-1α results in increased protein stability and transcriptional activity of HIF-1α, leading to increased PFKFB3 expression and abnormal glycolysis (32). HIF-1α further contributes to the glycolytic phenotype by upregulating PDK1 and LDHA while downregulating fatty acid oxidation, which is the primary energy source for healthy proximal tubular cells (33).

It is important to distinguish the evidentiary support for PFKFB3 versus PKM2. Jiang et al. directly proved causal regulation of PFKFB3 transcription by cGAS–STING–IRF3 through promoter-binding and functional rescue experiments (5). Although PKM2 has been described as a Warburg marker in multiple contexts, no kidney study has shown that specific genetic manipulation of PKM2 directly alters senescence-associated fibrotic phenotypes. In this review, available renal evidence positions PFKFB3 as the best-supported rate-limiting node linking cGAS–STING signaling to glycolytic amplification in the necroptosis–senescence axis; whether it is the sole or universal driver node across all metabolic reprogramming contexts awaits further genetic studies *in vivo*. PKM2, by contrast, should be regarded more as a downstream effector until kidney-specific genetic studies establish its causal role.

### 4.4 How the glycolytic microenvironment stabilizes and amplifies the senescent phenotype

Once glycolytic enhancement is established, it self-sustains and amplifies the senescent phenotype through multiple mechanisms. A classical positive-feedback loop forms among HIF-1α, TGF-β1, lactate, and LDHA. HIF-1α activates transcription of glycolytic genes; lactate stabilizes HIF-1α protein by inhibiting prolyl hydroxylase activity, bypassing normal oxygen-dependent degradation; TGF-β1, both a core SASP component and the master regulator of fibrosis, further upregulates HIF-1α and LDHA expression, while LDHA catalyzes lactate generation, providing substrate for HIF-1α stabilization (33). Once initiated, this loop operates continuously in a relatively stable hypoxic or pseudo-hypoxic microenvironment, locking senescent TECs into a high-glycolysis state.

PFKFB3-driven glycolysis is part of a wider metabolic disturbance in fibrotic kidneys. HIF-1α and Myc coordinate the shift from fatty acid oxidation to glycolysis in tubular, immune, and mesenchymal cells, while mitochondrial dysfunction and lipid accumulation further entrench the pro-fibrotic phenotype (34). Viewing glycolysis as one node within this integrated metabolic network clarifies why PFKFB3 inhibition can attenuate established fibrosis without necessarily being the sole initiator of disease (33).

Although the cGAS–STING–IRF3 axis initiates metabolic reprogramming, transcriptional amplification of SASP genes depends heavily on NF-κB. In renal and extra-renal contexts, NF-κB activation integrates inflammatory, metabolic, and senescent signals to sustain IL-6, IL-8, and CCL2 expression (35–37). Recent work in tumor-associated macrophages further shows that exosome-delivered CMTM4 can activate NF-κB, driving M2 polarization and secretion of TGF-β1 and CXCL12 (38). Because these findings derive from cancer models, any extrapolation to renal macrophages remains speculative; nevertheless, they raise a testable hypothesis that extracellular vesicles released from necrotic or senescent TECs may similarly engage macrophage NF-κB.

The signaling functions of glycolytic intermediate products further expand the pathological impact. Beyond lactate-mediated histone lactylation, succinate accumulation stabilizes HIF-1α in a hypoxia-independent manner by inhibiting PHD activity, promoting transcription of fibrosis-related genes (33). Enhancement of glycolytic flux is necessarily accompanied by decreased TCA cycle flux, leading to elevated NADH/NAD + ratios and reduced energy charge. This energy-stress state can activate AMP-activated protein kinase (AMPK) signaling, but the role of AMPK in fibrosis is bidirectional: short-term activation benefits cell survival, whereas long-term activation may affect metabolic adaptation through mTORC1 inhibition (33).

It must be cautiously noted that the causal status of glycolysis in TEC senescence has not been fully established. Existing evidence amply supports the proposition that glycolytic enhancement promotes maintenance of the senescent phenotype and sustained SASP secretion (5, 31), but direct evidence that glycolysis initiates or induces senescence remains limited. Glycolysis more likely plays the role of a sustainer rather than an initiator: once senescence signals push the cell into the senescent track, glycolytic enhancement stabilizes this state through positive-feedback mechanisms.

### 4.5 Toward a bidirectional positive-feedback loop: evidence and gaps

Together, the above mechanisms outline the individual arms of a putative bidirectional positive-feedback loop; simultaneous bidirectional causality in a single model remains to be established. In the necroptosis → senescence direction: membrane rupture releases DAMPs; mtDNA entering the cytoplasm of neighboring TECs activates cGAS–STING, driving PFKFB3 transcription and glycolytic reprogramming via IRF3; glycolytic enhancement produces lactate, which stabilizes pro-inflammatory and pro-fibrotic gene expression through H4K12la, while the HIF-1*α*/TGF-β1 loop locks in the metabolic phenotype, ultimately pushing the cell into stable senescence with sustained SASP secretion.

In the senescence → necroptosis direction, SASP factors secreted by senescent TECs can reciprocally activate necroptotic signals. Kandhaya-Pillai et al. found that TNF-*α*, a classical SASP component, induces autocrine signal transducer and activator of transcription (STAT) activation in senescent cells, and TNF-α is also one of the most potent inducers of necroptosis (39). The persistently elevated TNF-α concentration in the senescence microenvironment may maintain RIPK1 and RIPK3 in neighboring TECs near their activation threshold. Inflammatory factors such as IL-1α and IL-33 in SASP further amplify NF-κB activity via TLR and IL-1R signaling, and NF-κB is the common regulatory hub of both SASP and necroptosis signals (21).

A small number of senescent TECs can “infect” surrounding healthy cells through paracrine SASP action—termed paracrine senescence. Newly formed senescent cells expand SASP output, forming exponentially spreading senescence foci. Meanwhile, these cells or neighboring cells may undergo necroptosis under sustained inflammatory stress, releasing more DAMPs and mtDNA, again activating the cGAS–STING–PFKFB3–glycolysis axis. Metabolic reprogramming, especially PFKFB3-driven glycolytic enhancement, couples upstream death signals with downstream chronic senescent phenotypes, converting an acute injury event into a continuously progressive chronic fibrotic program.

One caveat: most evidence for this bidirectional loop derives from independent observations in different pathological models rather than longitudinal tracking of the same process. Necroptosis → senescence evidence comes mainly from acute injury models, whereas senescence → necroptosis evidence comes more from chronic senescence models. Simultaneously capturing real-time interactions between both directions in the same animal remains technically challenging.

## 5 Mechanisms of senescent tubular epithelial cell-driven interstitial fibrosis

**Figure 2:**
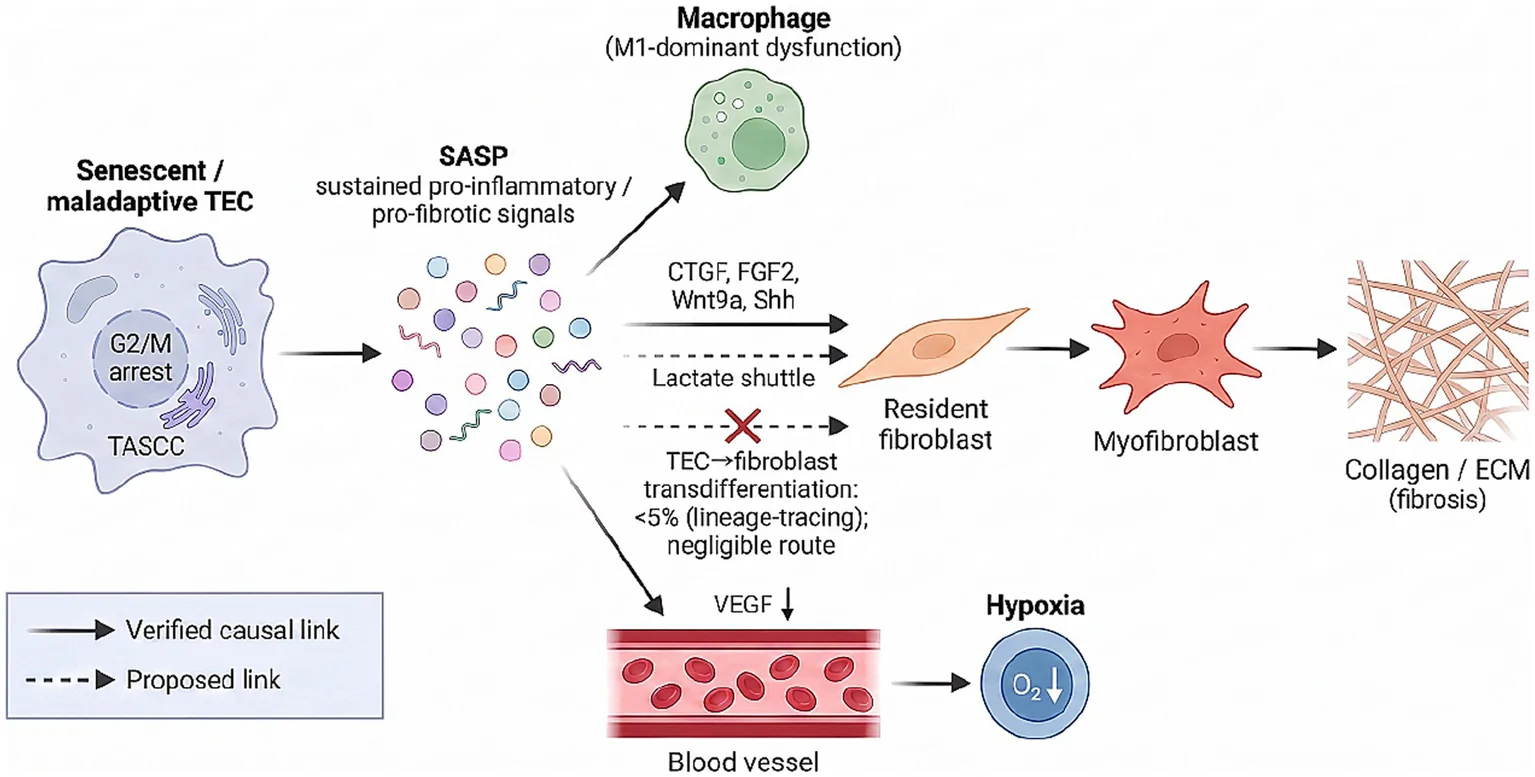
Cellular and molecular drivers of senescent TEC-mediated fibrosis. Solid arrows denote experimentally supported causal links; dashed arrows denote proposed or correlative links. Senescent/maladaptive tubular epithelial cells (TECs) establish G2/M cell-cycle arrest and assemble TOR–autophagy spatial coupling compartments (TASCC), which serve as structural platforms for sustained SASP production. SASP components recruit and polarize macrophages toward a dysfunctional M1-dominant state, while vascular endothelial growth factor (VEGF) deprivation drives capillary rarefaction and tissue hypoxia. Direct paracrine signaling (CTGF, FGF2, Wnt9a, and Shh) activates resident fibroblasts and drives their differentiation into collagen-secreting myofibroblasts, leading to extracellular matrix (ECM) deposition; metabolic coupling via lactate shuttling is proposed but less firmly established. Lineage-tracing studies indicate that TEC-to-fibroblast transdifferentiation is a negligible route (<5%), and maladaptive repair TECs function as signal hubs rather than fibroblast precursors, supporting the dominance of paracrine over transdifferentiation mechanisms.

Figure 2 depicts the cellular and molecular drivers. Senescent TECs have permanently exited the cell cycle, yet through persistent SASP secretion, they transmit pro-fibrotic signals that drive large-scale tissue remodeling from a small cellular substrate (21, 40).

### 5.1 SASP composition and kidney specificity

Classical markers of cellular senescence include stable cell-cycle arrest, elevated p16INK4a and p21CIP1, and enhanced SA-*β*-galactosidase activity, but the primary pathological driver is SASP. SASP is not a fixed profile; it shows high heterogeneity depending on senescence-inducing factors, cell type, and duration. López-Otín et al. noted that SASP composition can be divided into two functional waves: an early wave dominated by TGF-β, with anti-inflammatory and tissue-repair tendencies regulated by membrane-bound NOTCH1; as NOTCH1 declines, C/EBPβ drives a second phase dominated by pro-inflammatory cytokines and chemokines (21). This temporal feature is important in kidney pathology because senescent TECs may retain adaptive repair function in the early stage but transform into persistent pathological noise sources upon entering the chronic stage.

In CKD, core SASP components include interleukin (IL)-1β, IL-6, IL-18, transforming growth factor (TGF)-β1, tumor necrosis factor (TNF)-*α*, matrix metalloproteinase-2 (MMP-2), and plasminogen activator inhibitor-1 (PAI-1); these molecules constitute a pro-inflammatory, pro-fibrotic, and pro-vascular-injury secretory network (24, 40). In the kidneys of aged mice after IRI, transcript levels of Tgfb1, Il-6, Ctgf, and Serpine1 are significantly upregulated, suggesting that SASP is continuously activated during the AKI-to-CKD transition (24).

### 5.2 Paracrine activation of fibroblasts: from SASP to myofibroblasts

TGF-*β*1 remains the canonical driver of myofibroblast differentiation, acting through Smad3-dependent and Smad-independent mitogen-activated protein kinase (MAPK) pathways to induce alpha-smooth muscle actin (*α*-SMA) and collagen expression (41). However, renal fibrosis is not sustained by TGF-*β* alone. Connective tissue growth factor, platelet-derived growth factor, and epidermal growth factor form parallel pro-fibrotic networks that can maintain myofibroblast activation even when TGF-β signaling is partially suppressed (42, 43). This mechanistic redundancy helps explain why global TGF-β blockade has produced disappointing results in clinical trials.

The strongest human evidence comes from a 2024 study using conditionally immortalized CD10^+^ proximal tubular cells and PDGFRβ^+^ fibroblasts from human kidney tissue. Conditioned medium from senescent human PTECs was enriched for CCL2, CXCL1, GM-CSF, IL-6, and IL-8; when applied to fibroblasts, it induced myofibroblast differentiation with upregulated ACTA2, COL1A1, and FN1. In a cohort of more than 70 human renal biopsies, senescent TEC counts correlated positively with fibrosis severity and negatively with eGFR (44). This establishes a quantitative association between senescent TECs and fibrosis progression in human tissue.

In animal models, Li et al. used repeated low-dose cisplatin to show that BUMPT tubular cells develop typical senescence markers, and their supernatant—enriched in connective tissue growth factor (CTGF) and fibroblast growth factor 2 (FGF2)—promotes fibroblast proliferation; FGF2-neutralizing antibodies partially block this effect. shRNA knockdown of p16 inhibited senescence and attenuated fibrosis, establishing causality from both gain-of-function and loss-of-function perspectives. ABT-263 selectively killed senescent cells and reversed the pro-fibrotic phenotype (40).

Complex signaling loops amplify the effect. Luo et al. showed that Wnt9a accelerates tubular senescence; SASP-derived TGF-β1 activates fibroblasts, which secrete more Wnt9a, creating a self-reinforcing vicious cycle validated in multiple CKD models (45). Connexin 43-formed gap junctions and hemichannels also participate in direct TEC–fibroblast communication: Cx43-mediated hemichannel ATP release activates P2X7 receptors on neighboring cells, promoting epithelial phenotypic transition and fibroblast activation; blocking the Cx43–P2X7R axis attenuates interstitial fibrosis in diabetic kidney disease (46).

In diabetic kidney disease, senescent TECs secrete sonic hedgehog (Shh), which binds Ptch1 receptors on fibroblast surfaces, relieving Smoothened inhibition and activating Gli transcription factors to drive fibroblast proliferation and matrix synthesis. Shh knockdown or pharmacological inhibition both attenuate renal interstitial fibrosis in streptozotocin-induced diabetic mice (47). Lactate released by glycolytic TECs can be taken up by fibroblasts as a carbon source and acts as a signaling molecule to upregulate HGF, amplifying pro-fibrotic signaling (31, 33). TECs with sustained autophagy activate EGR1 through MAPK/ERK, inducing FGF2 secretion, which also activates fibroblasts (72). These findings describe a multi-mediator, multi-pathway parallel paracrine network.

### 5.3 G2/M arrest and TOR-autophagy spatial coupling compartments (TASCC): the pro-fibrotic secretory platform

Yang et al. established a causal relationship between epithelial cell G2/M arrest and fibrotic outcome through multi-model studies (48). After DNA damage or stress, some TECs fail to complete normal mitotic exit, arresting at G2/M. This state upregulates TGF-β1 and CTGF through c-Jun N-terminal kinase (JNK) and p53 pathways. JNK inhibitors or p53 inhibitors can partially rescue the fibrotic phenotype, suggesting G2/M arrest is not merely an accompanying marker but a structural foundation for pro-fibrotic function.

At the molecular level, TOR-autophagy spatial coupling compartments (TASCC) are observed in TECs arrested at G2/M. TASCC is a special subcellular structure formed by aggregation of lysosomes and autophagosomes in the perinuclear region; its core function is to provide energy and raw material support for sustained macromolecular synthesis and secretion. Atypical cyclin G1 promotes G2/M arrest and enhances TASCC formation; inhibiting TASCC reduces the proportion of G2/M-phase cells and attenuates renal fibrosis progression (73). TASCC is the secretory factory of senescent TECs, structurally coupling cell-cycle arrest with sustained SASP output.

### 5.4 Immune cell recruitment and chronic inflammation maintenance

CCL2 and CXCL1 systematically recruit monocytes/macrophages and neutrophils to infiltrate the renal interstitium. Unlike the acute inflammatory response dominated by M1 macrophages, chronic-phase polarization is imbalanced: M1 persists while M2 repair function becomes defective. This imbalance fails to clear senescent cells and further amplifies inflammatory signals (30, 40).

Macrophages are not a uniform effector population. Single-cell atlases of human and mouse kidneys distinguish resident macrophages from monocyte-derived subsets and have identified pro-fibrotic SPP1 + macrophages that spatially neighbor activated fibroblasts and promote myofibroblast activation (49, 50). Wnt/*β*-catenin signaling further polarizes macrophages toward an M2-like, pro-repair phenotype that paradoxically favors fibrosis (51). In addition, macrophage-to-myofibroblast transition provides a direct route by which infiltrating myeloid cells acquire collagen-producing capacity, particularly in obstructive and aging kidneys (52, 53, 74).

The chemokine CCL2 (monocyte chemoattractant protein-1, MCP-1) is a critical effector of macrophage recruitment. Proximal tubular epithelial cells are a major renal source of CCL2 after ischemic or toxic injury, and tubule-specific deletion of Ccl2 reduces macrophage infiltration and attenuates acute kidney injury (54). Once recruited, CCR2-expressing monocytes amplify inflammation and can undergo pro-fibrotic polarization, while CCL2 itself is upregulated by TNF-*α* in tubular cells via MAPK signaling (55). Beyond resident cells, extracellular vesicles have been shown to enhance CCL2 secretion by macrophages through mechanistic target of rapamycin (mTOR) activation in a cancer model (56); whether this mechanism operates in renal macrophages remains to be determined. In the kidney, the CCL2-CCR2 axis therefore functions as both a sensor of tubular damage and a feed-forward driver of chronic interstitial inflammation and fibrosis (57).

SASP vasoactive factors disrupt peritubular capillary endothelial cell integrity, leading to capillary rarefaction. Declining vascular density causes local hypoxia, inducing HIF-1α stabilization. HIF-1α directly upregulates TGF-*β*1 and various extracellular matrix genes, exacerbating fibrosis in the hypoxic microenvironment (33). A pathological network is formed between senescent TECs, macrophages, endothelial cells, and fibroblasts: TECs act as signal hubs to initiate and maintain the network, with fibroblasts as the ultimate effectors that cause excessive matrix deposition.

Endothelial injury introduces another level of crosstalk. Renal endothelial cells exposed to prolonged TGF-β and inflammatory stress can undergo endothelial-to-mesenchymal transition (EndMT), losing vascular markers and acquiring mesenchymal characteristics, thereby contributing to capillary rarefaction and tissue hypoxia (58). EndMT is functionally linked to metabolic reprogramming: loss of endothelial integrity exacerbates hypoxia, which in turn drives Myc-dependent glycolysis in tubular epithelial cells, creating a pernicious endothelial-epithelial feed-forward loop that parallels PFKFB3-driven glycolysis (59).

## 6 Controversies and unresolved questions

### 6.1 The identity crisis of epithelial–mesenchymal transition (EMT): do tubular epithelial cells truly transdifferentiate?

Classical EMT held that TECs transform into myofibroblasts. Rigorous genetic lineage tracing now shows epithelium-derived myofibroblasts account for less than 5% in some models or are undetectable (60, 61). The revised concept of “partial EMT” describes cells that downregulate epithelial markers and upregulate some mesenchymal genes while remaining anchored to the basement membrane, exerting pro-fibrotic effects through paracrine signaling rather than direct transformation. Abedini et al.’s “maladaptive tubules”—epithelial cells with highly active secretory function—are essentially the single-cell resolution molecular definition of partial EMT (3). Direct transdifferentiation is not necessary for fibrosis; paracrine activation by injured and senescent TECs is the dominant mechanism.

While epithelial contribution to myofibroblasts is limited, resident mesenchymal cells remain the dominant collagen-producing population. Single-cell and spatial multi-omics of human fibrotic kidneys have traced the differentiation trajectory from resident fibroblasts and perivascular progenitors to pathogenic myofibroblasts, revealing regional heterogeneity and a profibrotic niche that sustains ECM production (62–64). These observations do not rehabilitate EMT as a major mechanism; rather, they reinforce the concept that fibrosis is driven by diverse, context-dependent cellular sources and that effective therapy must target the mesenchymal-immune-epithelial ecosystem rather than a single transition program (1).

### 6.2 Necroptosis: pure villain or double-edged sword?

Necroptosis reduces injury and fibrosis when genetically or pharmacologically blocked, supporting its pathogenic role. But Pefanis et al. found that while RIPK1 or RIPK3 inhibition protects acutely and subacutely, chronic fibrosis is delayed rather than prevented (13). If necroptosis is merely the trigger, blocking it after the fibrotic program has started will not suffice. This tempers expectations for anti-necroptotic monotherapy in established CKD. In specific inflammatory contexts, necroptosis may exert tissue-protective effects by consuming excessively activated immune cells, but this “double-edged sword” hypothesis currently lacks direct experimental support in kidney models.

### 6.3 Senolysis versus senostasis: safety concerns

Senolytics such as ABT-263 and dasatinib plus quercetin attenuate fibrosis in models, and D + Q reduced systemic inflammation in a pilot clinical trial in patients with DKD (65). But senescent cells are not always pathological. Early after acute injury, transient senescence may aid basement membrane repair and inflammation resolution through TGF-*β* secretion. Early SASP is TGF-β-enriched and repair-oriented; only after senescence enters the chronic stage does SASP transform into an IL-6- and IL-8-dominated pro-inflammatory profile (21). Premature clearance may disrupt adaptive repair, leading to more severe tissue defects. The therapeutic window—acute repair versus chronic fibrosis—is central to senescence-targeting strategies.

### 6.4 Metabolic reprogramming: engine or passenger?

PFKFB3 inhibition or knockout attenuates fibrosis, meeting criteria for causal intervention. Yet all interventions were conducted after disease was established; no study has preventively blocked glycolysis before injury. Glycolysis likely maintains and amplifies senescence, but whether it initiates senescence remains unproven. TECs are among the most metabolically active cell types *in vivo*; moderate glycolysis may be an adaptive compensatory mechanism in acute hypoxia. Indiscriminate PFKFB3 inhibition could worsen the energy crisis in the acute phase, echoing a timing-dependent safety concern analogous to that of senolytic strategies.

## 7 Targeted therapeutic strategies

Current therapeutic strategies are compared in Table 2.

### 7.1 Necroptosis pathway inhibitors: from molecular targets to preclinical validation

Nec-1s protects the kidney from IRI even when given 15 min after reperfusion (13). GSK2982772, a highly selective RIPK1 inhibitor, has completed Phase I/II trials in psoriasis, rheumatoid arthritis, and ulcerative colitis (66, 67). Benzoxazolone compounds and primidone-based drug repurposing strategies have also shown potential to inhibit RIPK1–FADD interactions.

GSK’872 reduces tubular necrosis but induces apoptosis at high doses through off-target effects, limiting its therapeutic index. This safety bottleneck suggests RIPK3 may not be an ideal standalone target. At the most downstream point, MLKL is theoretically the most attractive target because inhibiting it blocks membrane lysis without interfering with potential physiological functions of upstream RIPK1 and RIPK3. However, no specific small-molecule MLKL inhibitor has entered preclinical investigation: the pseudokinase domain lacks a typical ATP-binding pocket, and activation depends on RIPK3-mediated phosphorylation and conformational change rather than kinase activity per se, making traditional kinase inhibitor design difficult to apply. No necroptosis pathway inhibitor is approved for kidney disease (8, 15).

### 7.2 Senescent cell clearance and state modulation: the double-edged sword of therapeutic window

ABT-263 (navitoclax), a BCL-2/BCL-xL inhibitor, clears senescent BUMPT cells, reduces CTGF and FGF2, and restores clonogenic capacity (40). Dasatinib plus quercetin reduced inflammatory markers in a pilot clinical trial in patients with DKD (65). Alternative senolytics such as fisetin and ABT-737 have shown similar effects in different models.

Senomorphic strategies—metformin and rapamycin—suppress SASP without killing cells: metformin inhibits NF-κB-dependent cytokine transcription through AMPK activation, while rapamycin reduces translation efficiency of IL-6 and IL-8 through mTORC1 inhibition. NAD + precursors improve mitochondrial function indirectly. The timing dilemma remains acute: clearance in the repair window may disrupt tissue recovery, whereas senostasis requires chronic dosing and may not fully silence secretion (21, 37).

### 7.3 Metabolic reprogramming intervention: from glycolysis blockade to mitochondrial protection

PFKFB3 blockade (3PO, PFK158) attenuates fibrosis in fibroblasts and myeloid cells (29, 30). HIF stabilizers such as FG-4592 help in acute ischemia but may worsen chronic fibrosis by promoting glycolysis. Lactate metabolism blockade—LDHA inhibition or MCT1/4 blockade—risks nephrotoxicity because complete blockade may interfere with energy supply in normal TECs.

Mitochondrial protection offers an upstream angle. Urolithin A, a natural mitophagy inducer, promotes PINK1/Parkin-dependent elimination of damaged mitochondria, which reduces cytosolic mtDNA and downstream cGAS–STING activation. A 2025 study showed that urolithin A significantly reduces cytosolic DNA accumulation and attenuates cGAS–STING-driven inflammasome activation in senescent cells (68). Szeto–Schiller peptides targeting the mitochondrial inner membrane and experimental mitochondrial transplantation therapy have also shown renal protective potential but remain distant from clinical translation.

### 7.4 Traditional Chinese medicine: adjunctive multi-node modulation and the standardization gap

The following botanical and formula studies report broad anti-inflammatory, anti-oxidative, or general anti-fibrotic effects in renal fibrosis models; most have not been validated against the specific mtDNA–cGAS–STING–PFKFB3 axis discussed above. Pending mechanism-focused investigations, these agents are more appropriately positioned as adjunctive therapies rather than targeted interventions against the metabolic bridge. Representative examples include astragaloside IV (Astragalus membranaceus), emodin and rhein (*Rheum palmatum*), tanshinone IIA (*Salvia miltiorrhiza*), and formulas such as Shenkang Injection and Liuwei Dihuang pills; these have been reported to modulate phosphatidylinositol 3-kinase (PI3K)/protein kinase B (AKT), AMPK/mTOR, TGF-*β*/Smad, NF-κB, and nuclear factor erythroid 2-related factor 2 (Nrf2) signaling in renal injury models (69). Cordyceps sinensis preparations have also shown multi-pathway effects in diabetic kidney models (69).

Mechanistically, these botanical agents converge on inflammatory and fibrotic hubs discussed above—such as NF-κB, TGF-β1/Smad, and oxidative stress—rather than directly targeting the mtDNA–cGAS–STING–PFKFB3 metabolic axis. This broad multi-node activity aligns with the concept of network remodeling, but standardization of herbal extracts, batch-to-batch consistency, and rigorous clinical validation remain major translational barriers. TCM may therefore serve more appropriately as an adjunctive rather than a replacement therapy until quality control and RCT evidence improve.

## 8 Translational barriers and future directions

### 8.1 Target selectivity and delivery precision: the preclinical–clinical gap

RIPK1 and PFKFB3 have physiological roles beyond the kidney; systemic inhibition risks infection, anemia, and impaired angiogenesis. This “narrow therapeutic window” problem is particularly prominent in the kidney, a metabolically active organ with abundant blood perfusion where high renal drug clearance means the kidney obtains higher concentrations but also exacerbates non-target cell exposure. Kidney-targeted delivery may solve this dilemma. Passive targeting using 75–100 nm nanoparticles can traverse the fenestrated glomerular endothelium and accumulate in the mesangial region (75). Active targeting through megalin receptor-mediated proximal tubular uptake can deliver drugs directly to the core pathological cell population. CD44 and integrin αvβ3 markers can guide precise drug release in matrix deposition regions. Stimulus-responsive prodrug strategies activated under low pH or high ROS improve selectivity for the pathological microenvironment. However, these strategies remain largely at the concept-validation stage; pharmacokinetics in large animals, long-term biocompatibility, and scaled production feasibility have not been systematically evaluated. For now, molecular design optimization is more realistic than delivery breakthroughs (2).

### 8.2 Biomarker absence: the bottleneck for monitoring dynamics and assessing response

Serum TGF-β1, CTGF, MMP-2, and MCP-1 correlate with progression but cannot distinguish reversible fibrosis from irreversible scarring and cannot reliably predict therapeutic response at the individual level. This defect forces reliance on surrogate endpoints such as eGFR, yet eGFR fluctuation is influenced by multiple confounding factors, and its correspondence with histological fibrosis degree is not always linear.

Liquid biopsy technologies provide potential breakthroughs. Urinary single-cell transcriptomics can noninvasively obtain gene expression from TECs and immune cells; exosomal miRNA profiling reflects tissue-level microRNA changes; and circulating cfDNA methylation patterns may carry epigenetic signals of kidney-specific injury. Serum levels of the collagen V α1 chain (Col5a1) were quantitatively correlated with the severity of fibrosis and predicted a response to the integrin αvβ3 inhibitor Cilengitide in a 2025 preclinical (murine) study published in Science Translational Medicine (70). This provides a concrete stratification marker, demonstrating the feasibility of biomarker-guided precision therapy. But standardization, cost-effectiveness, and regulatory approval remain hurdles. Before validated biomarkers become available, renal biopsy remains the only reliable means of assessing fibrosis dynamics, and its invasive nature severely limits repeat application.

### 8.3 Trial design lessons: therapeutic window and molecular stratification

The history of renal fibrosis drug development provides a case library for translational medicine (Table 3). Lademirsen (miR-21 antisense) was terminated early in Phase II in Alport syndrome, probably because miR-21 sits upstream of a fibrogenic network whose inhibition can be bypassed by compensatory pathways. Fresolimumab (anti-TGF-*β*) failed to show renal function protection over placebo in focal segmental glomerulosclerosis and diabetic kidney disease, illustrating that global TGF-β suppression disrupts both pro-fibrotic and homeostatic TGF-β signaling. Bardoxolone methyl was halted in the Phase III BEACON trial because of increased cardiovascular events and mortality, likely reflecting off-target vascular effects rather than anti-fibrotic failure per se. Cilengitide (integrin αv*β*3 inhibitor) showed efficacy only in a Col5a1-high subgroup, demonstrating that molecular stratification can rescue a positive signal even when the overall trial is negative. Collectively, these failures share several lessons.

The first lesson is the inherent limitation of single-target strategies in complex fibrotic networks. Fibrosis is driven by synergistic dysregulation of multiple cell types and signaling axes. Simply blocking TGF-β or inhibiting miR-21 may trigger compensatory reorganization of the pathological network—upregulation of other pro-fibrotic pathways may offset the effect. The cardiovascular toxicity of bardoxolone may relate to its broad antioxidant and anti-inflammatory effects disturbing vascular homeostasis.

The second lesson is the importance of therapeutic windows and molecular stratification. The same intervention strategy may produce markedly different effects in the acute, subacute, and chronic phases. HIF stabilizers may exert protective effects in acute ischemia by promoting adaptive metabolism, but in chronic fibrosis may exacerbate glycolysis dependence and matrix deposition. If clinical trials do not stratify subjects according to disease stage or molecular subtype, positive and negative effects may cancel each other out, leading to overall negative results. The Col5a1-stratified response to Cilengitide illustrates that biomarker-guided enrichment can rescue positive signals in heterogeneous CKD populations (70).

**Figure 3:**
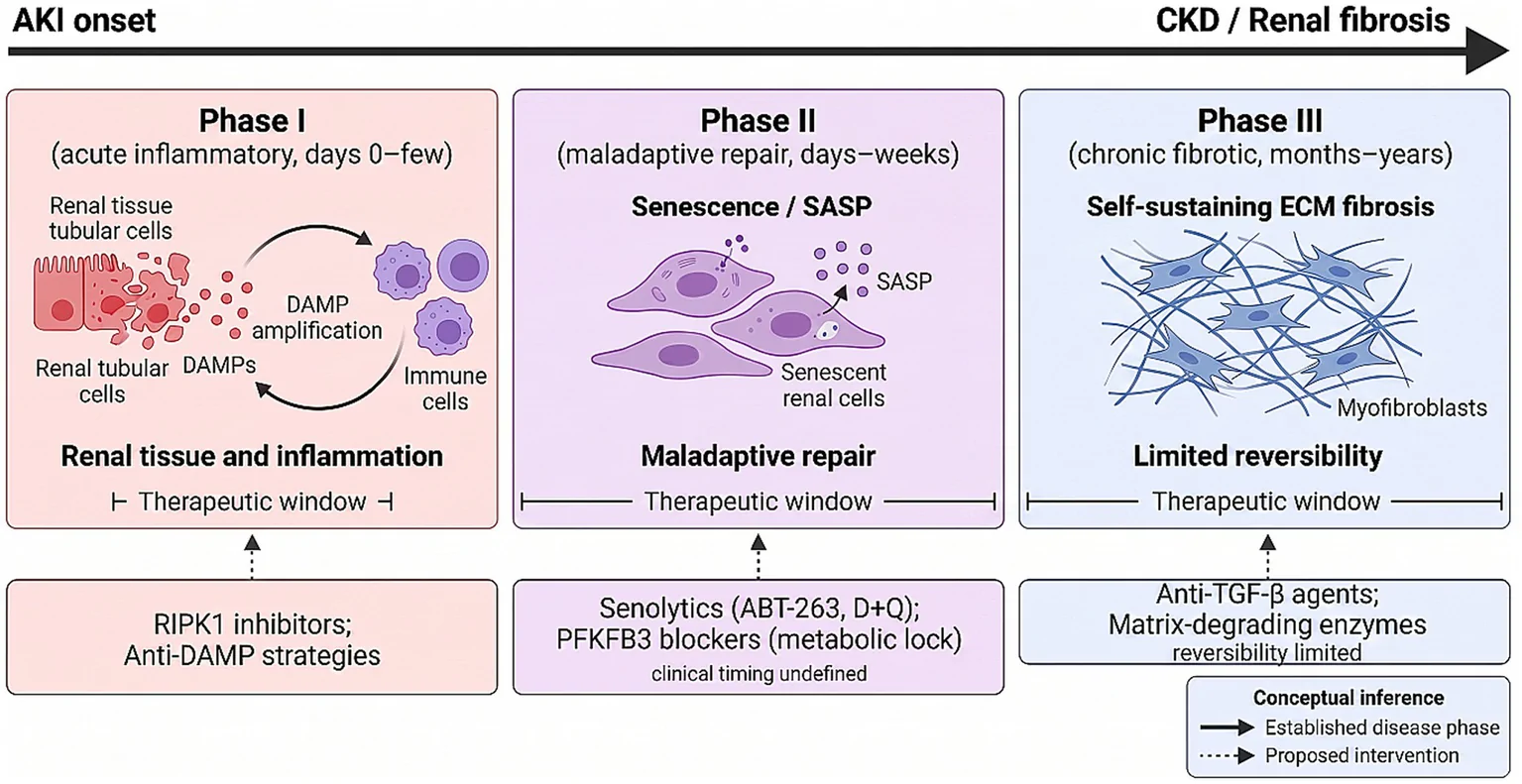
Time-stratified intervention model for renal fibrosis. The horizontal timeline (solid arrows) represents the established AKI-to-CKD transition; dashed arrows/boxes indicate proposed stage-specific interventions that remain largely supported by preclinical models. Phase I (acute inflammatory phase, days the first few days): Necroptosis-driven DAMP release and innate immune activation dominate. RIPK1 inhibitors and anti-DAMP strategies may have the greatest benefit here, but excessive inflammation suppression may weaken repair signals. Phase II (maladaptive repair phase, subacute days–weeks): TECs enter senescence, SASP establishes, and fibroblasts gradually activate. Senolytics and PFKFB3 inhibitors are proposed to target the metabolic lock that stabilizes the fibrotic program, yet their clinical timing remains undefined. Phase III (chronic fibrotic phase, months–years): Sustained extracellular matrix deposition and scar maturation characterize this phase. Anti-TGF-β agents and matrix-degrading enzymes are needed, but reversibility has significantly narrowed. The width of each arrow reflects the estimated therapeutic window; the color gradient from red to blue indicates the shift from inflammatory to fibrotic pathology.

A proposed time-stratified intervention model is shown in Figure 3. Phase I (acute inflammatory, the first few days): necroptosis-driven DAMP release and innate immune activation dominate. The intervention goal is to suppress excessive cell death and inflammatory storms, protecting reversibly injured TECs. RIPK1 inhibitors and anti-DAMP strategies may have the greatest benefit here, but excessive inflammation suppression may weaken repair signals.

Phase II (maladaptive repair, subacute days–weeks): TECs enter senescence, SASP establishes, and fibroblasts gradually activate. The focus shifts from death suppression to senescence modulation and metabolic reprogramming: senolytics, PFKFB3 inhibition, and mitochondrial protection may be most effective. Intervention success or failure here largely determines whether fibrosis locks into an irreversible course.

Phase III (chronic fibrotic, months–years): sustained ECM deposition and scar maturation characterize this phase. Upstream death and senescence signals may have completed their trigger function, and the fibrotic program has acquired self-sustaining capacity. Anti-TGF-*β* agents and ECM degradation promoters are needed, but reversibility has significantly narrowed.

No universal anti-fibrotic drug fits all CKD stages. Future trial designs must incorporate temporal stratification, grouping patients by disease stage, fibrosis activity markers, and aetiological subtype rather than enrolling all CKD patients into a unified cohort.

### 8.4 Future directions: from single-molecule targets to network remodeling

Multi-omics integration—single-cell and spatial transcriptomics, proteomics—provides the technical foundation for system-level fibrotic atlases. These atlases can reveal hidden connections between known pathways and discover novel regulatory nodes inaccessible to traditional methods (3, 63). AI-driven network pharmacology provides tools for optimizing integrated Chinese and Western medicine strategies. Machine learning algorithms analyzing large-scale compound–target–disease networks can predict multi-target action patterns and identify synergistic component combinations. This converges methodologically with TCM’s holistic regulation philosophy (69).

At the precision medicine level, biomarkers such as Col5a1 mark a shift from one-size-fits-all to stratified therapy. Future trials should base enrollment and efficacy assessment on molecular characteristics rather than merely clinical phenotypes. The most effective strategy may be temporal combination therapy: necroptosis inhibitors in the acute phase, senolytics and metabolic modulators in the subacute phase, and anti-fibrotics plus ECM degradation promoters in the chronic phase. Successful implementation depends on biomarker development and kidney-targeted delivery technology (2). Network remodeling approaches that integrate liquid biopsy staging, targeted delivery, and multi-omics modeling may further improve spatiotemporal precision (71).

## 9 Conclusion, recommendations and outlook

### 9.1 Key conclusion

This narrative review systematically describes the molecular crosstalk between necroptosis and cellular senescence in CKD fibrosis. Three conclusions stand out. First, necroptosis and TEC senescence form a bidirectional positive-feedback loop through the mtDNA–cGAS–STING–PFKFB3–glycolysis axis. Second, PFKFB3-mediated glycolysis acts as a metabolic sustainer and amplifier—and potentially a bridge—connecting acute cell death with chronic fibrosis; its role as an initiator remains unproven. Third, existing strategies show preclinical promise, but clinical translation is constrained by target selectivity, biomarker absence, and imprecise therapeutic windows; future success depends on shifting from single-molecule inhibition to multi-node network remodeling.

### 9.2 Recommendations for the field

1. Establish longitudinal AKI-to-CKD models tracking necroptosis and senescence at single-cell resolution to clarify temporal causality.
2. Develop kidney-specific MLKL functional probes to test whether non-necroptotic MLKL functions exist in TECs—a question currently only studied in liver and neural models.
3. Incorporate explicit temporal stratification in senolysis trials to distinguish acute repair from chronic fibrosis phases.
4. Advance TCM quality control standardization, combining HPLC fingerprinting with bioactivity detection.

### 9.3 Outlook

In the short term (within 5 years), the realistic goal is optimizing combinations of existing standard therapies and exploring RIPK1 inhibitors in transplant ischemia–reperfusion injury. In the medium term (5–10 years), senolytic trials may expand to larger Phase II studies, and Col5a1-stratified strategies may enter clinical testing. In the long term (beyond 10 years), temporal combination therapy tailored to individual molecular profiles may become the new standard—provided trial design philosophy shifts from pursuing universal single-drug efficacy to embracing precision medicine that acknowledges disease heterogeneity and temporal dynamics.
